# Diagnostic sensitivity and specificity of dynamic three-dimensional CT analysis in detection of cam and pincer type femoroacetabular impingement

**DOI:** 10.1186/s12891-020-3049-3

**Published:** 2020-01-16

**Authors:** Maarten A. Röling, Nina M. C. Mathijssen, Rolf M. Bloem

**Affiliations:** Department of Orthopaedic surgery, Reinier de Graaf Hospital, Reinier de Graafweg 3-11, 2625 AD Delft, the Netherlands

**Keywords:** Femoroacetabular impingement, Diagnostics, CT, Radiograph

## Abstract

**Background:**

Cam and pincer-type morphologies can cause femoroacetabular impingement syndrome (FAI) and can be measured on plain radiographs using the alpha angle and the center edge angle. As an addition to plain radiographs and to assess femoroacetabular impingement, it is possible to visualize the interplay of the acetabular and femoral morphology by means of dynamic three-dimensional simulation of hip joint. Therefore, the objective of this study is to compare alpha angles and center edge angles on plain radiographs with the dynamic computerized tomography (CT) analysis in patients with complaints of femoroacetabular impingement.

**Methods:**

All patients from our prospective cohort from 2012 to 2015 who underwent radiographs and a dynamic CT analysis for FAI were selected. Cam type morphologies were measured with the alpha angle and pincer type morphologies with lateral center-edge angle on radiographs and with CT analysis. The dynamic CT analysis also calculated position and size of impingement of femur and acetabulum. Intra-operative assessment was used to confirm impingement. Sensitivity, specificity and predictive values were calculated compared with respect to the intra-operative assessment.

**Results:**

A total of 127 patients were included. 90 cam morphologies and 45 pincer morphologies were identified intra-operatively.

The sensitivity and specificity for cam morphology measured with radiographs was 84 and 72% compared to 90 and 43% with three dimensional dynamic analyses. The sensitivity and specificity for pincer morphology measured with radiographs was 82 and 39% compared to 84 and 51% with three dimensional dynamic analyses.

**Conclusions:**

Diagnostic accuracy is comparable in three-dimensional dynamic analysis of CT scans and radiographs representing FAI caused by cam or pincer type morphology.

**Level of evidence:**

IV

## Background

Femoroacetabular impingement (FAI) syndrome is a well-known cause of hip related pain in athletes and active persons [1]. FAI syndrome can be caused by cam and pincer type morphologies [2]. A cam type morphology is caused by an osseous deformity of the femoral head-neck contour, an overgrowth of bone, which can impinge with the acetabular rim during flexion and rotation of the hip. A pincer type morphology is an over-coverage of the acetabulum, which can be focal, and can also cause impingement of the joint. Both morphologies can cause damage in the hip joint, which might result in pain and possible degeneration of the hip joint. Resection of these bony morphologies with hip arthroscopy can relieve the impingement and the pain caused by it [3]. It might also prevent further degeneration of the hip joint [4]. Identification of the exact location of these morphologies is essential in order to be able to adequately treat the impingement. Intra-operative assessment of typical labral and cartilage lesions associated with cam or pincer type lesions seems the optimal diagnostic method. For cam type impingement damage to the anterosuperior acetabular cartilage with separation between the labrum and cartilage was identified. During flexion, the cartilage is sheared off the bone by the non-spherical femoral head while the labrum remains untouched. This typical damage caused by a cam morphology is a chondro-labral disruption and a progressive chondral delamination: a so-called wave sign. A cam type morphology, the asphericity of the femoral head, was identified in the peripheral compartment after release of the traction.

Damage from a pincer morphology causes an extensive degeneration of the labrum and the adjacent chondral surface. The cartilage damage is located circumferentially and includes only a narrow strip. During movement the labrum is crushed between the acetabular rim and the femoral neck causing degeneration and ossification of the labrum.

The intra-operative assessment, however, should not be used as a diagnostic tool alone, because of its invasive nature. Initial clinical evaluation is mainly done with plain radiographs [5] using the alpha angle [6] to detect cam morphology and lateral center-edge (LCE) angles, crossover signs and other modalities to detect pincer morphology [7]. However, plain radiographs have limitations due to their two-dimensional visualization of this three-dimensional process. The sensitivity for alpha angles varies widely but is described as high, up to 91% on Dunn views. Described inter and intra observer reliability varies also, with intraclass correlation coefficient (ICC) 0.43 for the alpha angle [8] and ICC 0.88 for LCE angles [9] The sensitivity for LCE angles is 84% [10]. These measurements seem quite reliable, but still lack information about the actual impinging moment of the morphologies. The presence of a cam or pincer morphology does not define an impinging hip, it only defines a deviating morphology. Imaging modalities like computed tomography (CT) or magnetic resonance imaging (MRI) might be a better diagnostic option for this three-dimensional process, compared to radiographs.

Dynamic CT analysis was validated for use in FAI analysis in a cadaver model [11]. With dynamic CT analysis, a three-dimensional model of the hip is made to detect the area of femoroacetabular impingement. The software calculates the angles defining cam or pincer type morphologies and it also creates a dynamic analysis to identify impingement of hip and acetabulum within a pre-defined range of motion [12–14] of the hip joint.

However, no clinical studies regarding dynamic CT analysis in patients suspect for FAI syndrome have been performed. Therefore, the objective of this study is to compare alpha angles and center edge angles on plain radiographs with the angles measured on dynamic CT analysis in patients with complaints of FAI syndrome. We compared the sensitivity, specificity and predictive values of radiographs with dynamic CT-analyses with respect to the intra-operative assessment.

It was hypothesized that dynamic CT analysis has a higher sensitivity and specificity in representing the impinging cam and pincer type morphology compared to the radiographs.

## Methods

**Fig. 1:**
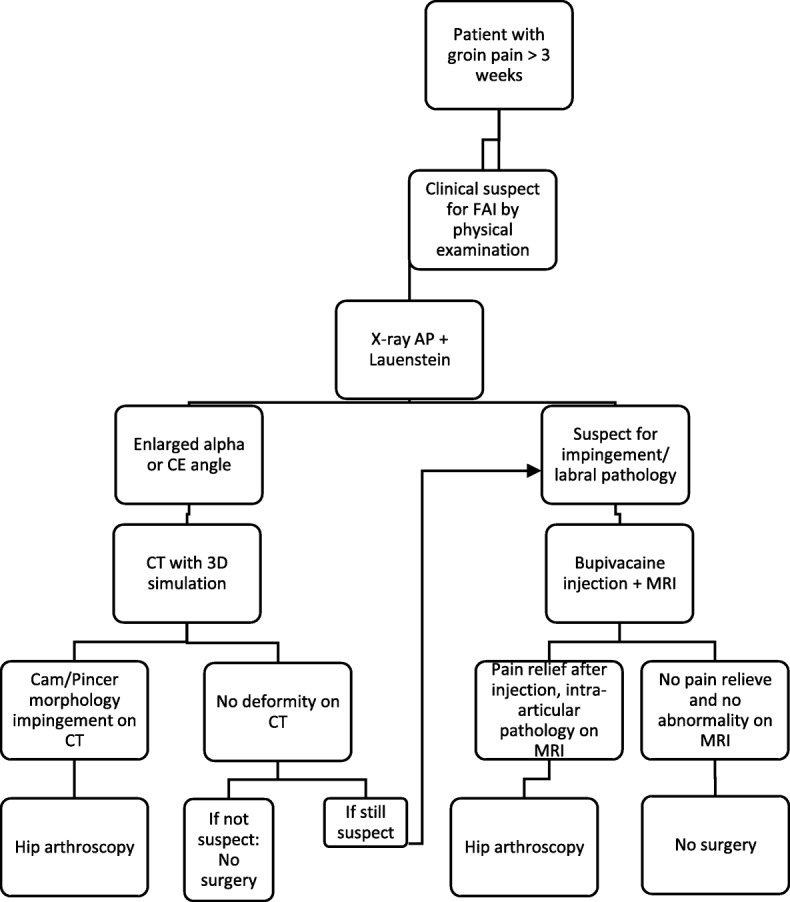
diagnostic flowchart femoroacetabular impingement

The present study used data from an ongoing prospective registry in our hospital. We selected all patients who underwent radiographs and dynamic CT analysis for FAI diagnostics and who were operated on between 2012 and 2015. Inclusion criteria for the prospective registry were: diagnosed with FAI syndrome (i.e. evident clinical signs of femoroacetabular impingement [15], positive clinical assessment with positive tests specific for FAI [15] flowchart Fig. 1), age 18–65, managed conservatively first (with strengthening physiotherapy for at least three months, lifestyle changes and non-steroid anti-inflammatory drugs), suitable for surgery (after consultation of the anesthesiologist for any contra-indications for surgery) and patients have to be willing to participate. Exclusion criteria are age < 18 or > 65, prior hip arthroscopic surgery patient history and/or pathological fractures due to metastatic disease.

All patients were operated in our peripheral teaching hospital (location blinded). All patients signed informed consent to participate and to publish. The local Medical Ethics Committee decided that the study did not fall under the scope of the Medical Research Involving Human Subject Act because of the minimal burden for patients in comparison to regular care (METC nr 12–083). The data were retrospectively analyzed.

### Radiographic measurements

Radiographic antero-posterior (AP) and Lauenstein images were made when patients were included. Radiographs were performed using standardized techniques in the supine position as described by Clohisy et al. [16]. AP pelvis radiographs were performed with the legs 15° internally rotated with the beam centered between the superior anterior iliac spine and symphysis pubis. The Lauenstein views were performed the hip in 30–40° of flexion and 45° of abduction with the heel a rest against to contralateral medial side of the knee.

**Fig. 2:**
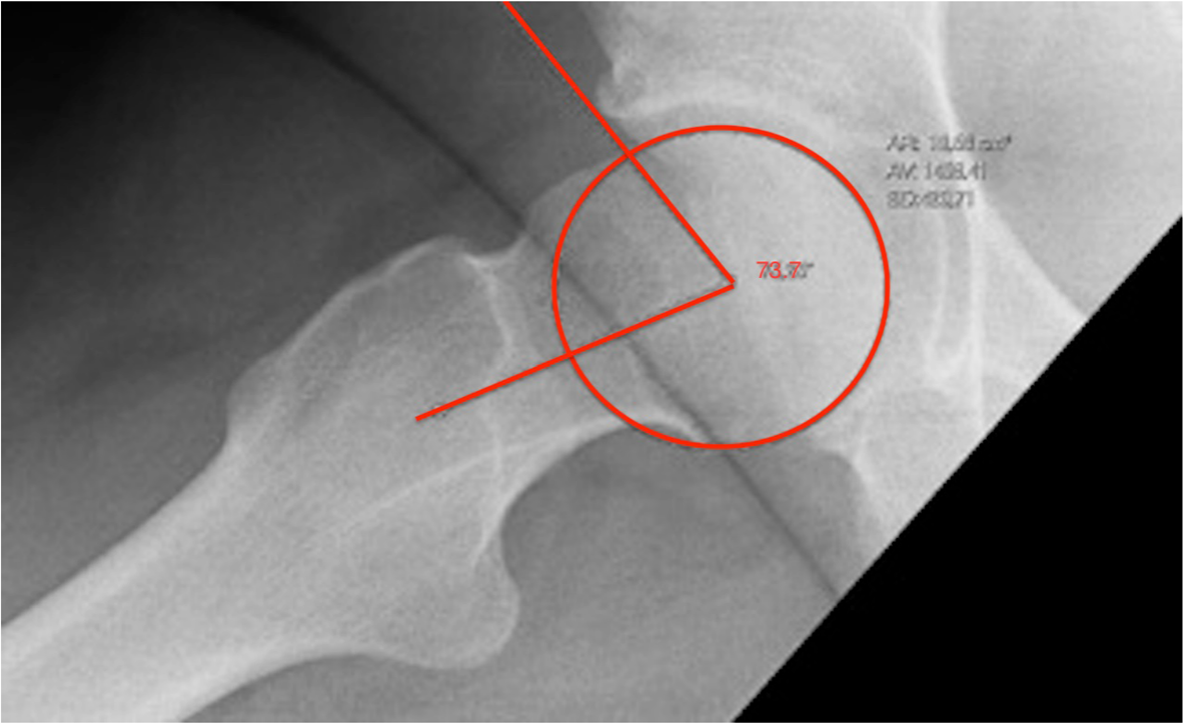
example of an alpha angle measured on a Lauenstein x-ray

A cam type morphology was measured on a Lauenstein radiograph by measuring the alpha angle. The angle is measured between two lines: a line from the center of the femoral head to the point where the radius of the femoral head exceeds a perfect circle drawn around the femoral head, and the line drawn from the center of the femoral head to the center of the femoral neck. An angle larger than 60° was considered an enlarged alpha angle and an indicator of cam morphology indicating FAI [6, 17, 18] Fig. 2.

A pincer type morphology is measured on an AP pelvic radiograph with the lateral center edge (LCE) angle. This is the angle between a line vertical to the center of rotation of the femoral head and the lateral edge of the acetabulum Fig. 4. An LCE angle larger than 33° was considered enlarged and an indicator of pincer morphology indicating FAI [10, 19]. All x-rays were interpreted by an independent radiologist who made a report in the patient file and by one of the researchers (MAR).

### Dynamic CT analysis

The CT scans of the pelvis were performed with a standardized protocol. CT scans were performed at the department of Radiology using a second-generation dual source multi-detector spiral CT scanner (SOMATOM Definition Flash, Siemens Healthcare) AG, Erlange, Germany) with a tube voltage of 80 Kv and an effective mAs-value of 3140. Scan timer per CT scan was approximately 30 s. All patients were scanned in the standard anatomic axial plan orientation and were reconstructed with an effective slice thickness of 1.0 mm and a sharp reconstruction kernel (B75s). Multi-planar reconstruction was performed (image pixel size 0.265).

**Fig. 3:**
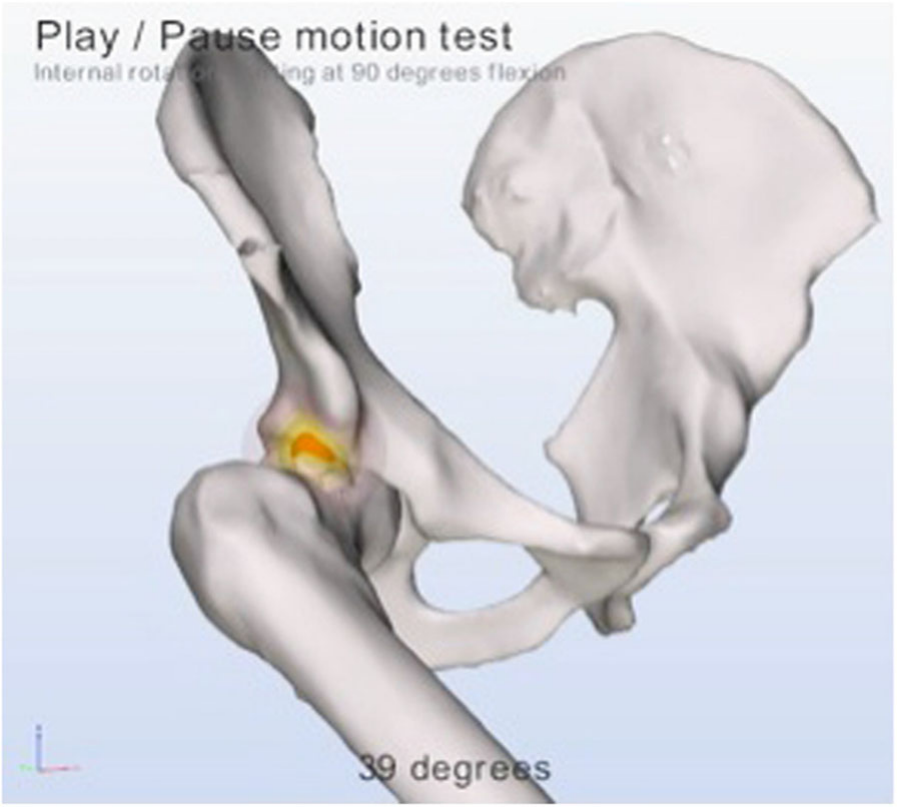
a simulated internal rotation and flexion of the right hip joint with simulated impingement as represented by the software

The dynamic analysis of the hip joints was made with proprietary software of Clinical Graphics® [20] which uses the coordinate systems as described in the recommendations of the International Society of Biomechanics and the equidistant method described by Puls et al. to simulate translation of the femoral head [21]. The software was previously validated in 2015 [11]. Figure 3 is an example of a cam type morphology causing impingement during simulated internal rotation as provided by the software. If kinematic motion is limited, the software reports the depth and location of the impingement and exact location of the type morphology, a dynamic movement analysis with exact impinging locations, an alpha angle (at seven positions from nine till three o’clock), a center edge angle (at three positions; 11, 12 and one o’clock positions) and a reproduction of the unlimited range of motion [11, 20]. An impingement is only detected within normal range of motion of the hip joint, according to relevant literature, which is discussed in the validation study [9].

The Dynamic CTs were interpreted by the software company who provides the software and dynamic analyses (Clinical Graphics), for which a detailed report of the analysis was made. Their reports and scans were also interpreted by one of the researchers (MAR).

### Surgery

All patients underwent hip arthroscopy for treatment of the FAI. Intra-operative images with fluoroscopy were used to determine if the hip joint was adequately widened with traction and whether the impinging areas were adequately resected.

Examination of the joint and operative technique was performed in accordance with Bond et al. [22]. The intra-operative assessment contained documentation of intra-articular damage to cartilage or labrum, caused by impingement, as described by Beck et al. [23]. This contained inspection of the central and the peripheral compartment. Damage to the anterosuperior cartilage of the acetabulum, a chondral delamination, separation of the labrum and cartilage, degeneration of the labrum and chondral surface on the femoral head, pincer morphologies caused by a bony edge of the acetabulum, cam morphologies on the femoral head neck junction or signs of herniation pits on the femoral head neck junction were identified and recorded in the patient file. Locations and types of lesions were recorded in the patient file.

Impingement can be proven by identifying and recording such typical lesions to the hip joint.

These intra-operative signs of impingement were afterward used as the golden standard for impingement, to compare with the pre-operative diagnostics methods of x-rays and dynamic analyses.

### Statistics

A cam type morphology suspect for FAI was defined as an alpha angle > 60° measured on Lauenstein radiographs.

The presence of a cam type morphology on dynamic CT analysis was defined as an osseous impinging area on the anterolateral side of the collum, due to the asphericity of the femoral head. This was highlighted by the software during the simulated range of motion within values of normal hip motion. Example Fig. 3.

A pincer type morphology was defined as an LCE angle > 33° measured on AP radiographs.

The presence of a pincer type morphology on the dynamic CT analysis was defined as an osseous impinging area on the anterior, lateral or posterior wall of the acetabulum, highlighted by the software during the simulated range of motion within values of normal hip motion.

The intra-operative assessments with identification of impinging cam and/or pincer type morphologies, were considered as the gold standard for impingement.

Sensitivity, specificity, positive-predictive-values (PPV) and negative-predictive-values (NPV) were calculated.

Software of Microsoft excel for MAC 2011, version 14.7.7. Was used for the calculations

## Results

A total of 127 patients were selected for analysis. Table 1 presents demographic data and intra-operatively registered morphologies of this cohort.

**Table 1:** demographic characteristics of the patient population

Patients	*N* = 127
Male/Female	78/49
Age	37.5 (18–65)
Years of complaints	3.6 (1.0–30)
Alpha angle on X-ray	66° (39°-96°)
Lateral center edge angle on X-ray	38° (25°-75°)
Deformities intra-operative	
Cam	90 (71%)
Pincer	45 (35%)
Combined cam and pincer	29 (23%)
Labral tears	83 (65%)

A total of 90 cam morphologies and 45 pincer morphologies were diagnosed intra-operatively. In 29 patients, a mixed-type morphology was present. The average alpha angle measured on a Lauenstein radiographs was 66°. The average LCE angle measured on an AP radiograph was 38°.

The alpha angle on radiographs indicated FAI due to a cam morphology in 86 patients (alpha > 60°), of whom 76 showed signs of an impinging cam morphology with the intra-operative assessment.

The dynamic CT analyses showed impinging cam morphologies in 102 patients, of whom 81 showed signs of impinging cam morphology with the intra-operative assessment.

The LCE angle on radiographs was > 33° in 87 patients, of whom 37 showed signs of an impinging pincer morphology with the intra-operative assessment.

The dynamic CT analyses showed impinging pincer morphologies in 78 patients, of whom 38 showed signs of impinging pincer morphology with the intra-operative assessment.

The sensitivity, specificity, PPV and NPV are reported in Tables 2 and 3

**Table 2:** sensitivity, specificity, PPV^a^ and NPV^b^ for cam type morphology comparing radiographs and dynamic CT scans with the per-operative assessment

	Sensitivity	Specificity	PPV^a^	NPV^b^
Alpha angle on X-ray	84%	72%	88%	63%
Dynamic CT impingement	90%	43%	79%	64%

^a^Positive predictive value

^b^Negative predictive value

**Table 3:** sensitivity, specificity, PPV^a^ and NPV^b^ for pincer type morphology comparing radiographs and dynamic CT scans with the per-operative assessment

	Sensitivity	Specificity	PPV^a^	NPV^b^
LCE angle on X-ray	82%	39%	43%	80%
Dynamic CT impingement	84%	51%	49%	85%

^a^Positive predictive value

^b^Negative predictive value

## Discussion

The objective of this study was to compare sensitivity, specificity and predictive values of radiographs with dynamic CT-analyses, with respect to the intra-operative assessment. For cam type morphology, the dynamic CT-analyses has higher sensitivity and NPV, but a lower specificity and PPV compared to radiographs. For pincer type morphology, only small differences could be observed in favor of the dynamic analysis. The use of a three-dimensional dynamic analysis of CT scans could be a useful tool for surgeons in their preoperative assessment, but the diagnostic value is comparable with the sensitivity and specificity of radiographs.

The use of an alpha angle to define cam type morphology [17, 24] is debatable. Some authors have described sensitivity up to 91% for an alpha angle on Dunn views [7, 10, 17, 25]. Our results show sensitivity for an alpha angle > 60° of 84%. Variations in diagnostic accuracy might be due to variations in the used alpha angles, different sizes of patient cohorts and differences in intra-operatively used assessment of impingement damage to the joint. However, the use of radiographs gives no information of the position of the impinging area and the amount of bone needed to resect, to resolve the impingement.

**Fig. 4:**
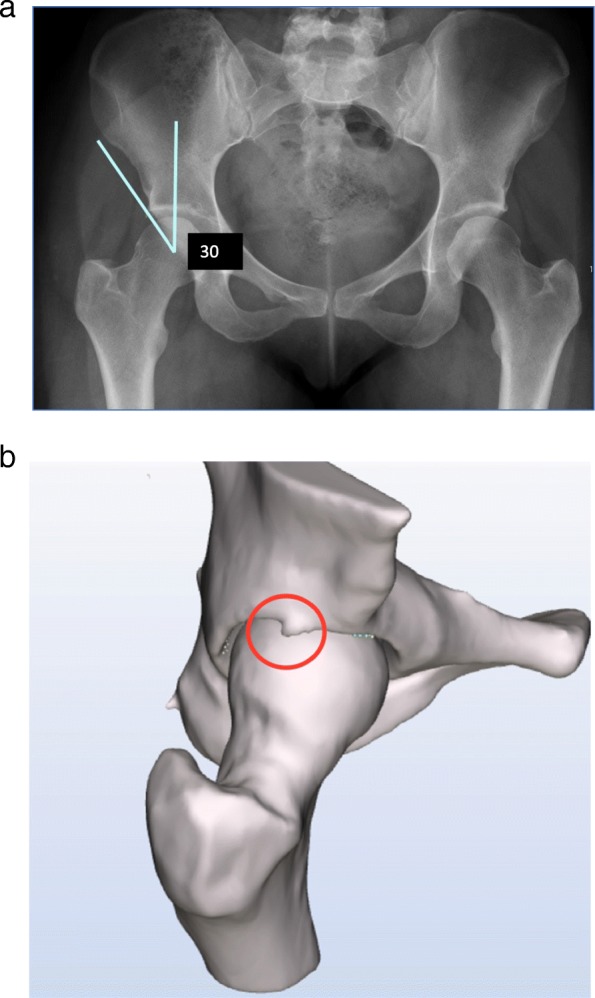
**a** example LCE angle within normal limits, no detection of a pincer type morphology. **b** the same right hip joint seen from sagittal plain: a single osteophyte causing a pincer type morphology outside the coronal plain and impinges in abduction and flexion

A cam type morphology causes asphericity of the femoral head. Whether this asphericity causes FAI syndrome is defined by several factors, for example the version of the femoral neck, the shape of the acetabulum and the actual size and depth of the morphology. A relatively deepened acetabulum, protrusio acetabuli or retroversion of the collum and/or acetabulum, combined with a minor enlarged alpha angle cam type morphology might cause FAI syndrome. Representing the cam morphology only by the alpha angle gives no information about the shape of the acetabulum and the movement of the hip joint causing the impingement. We identified several morphologies outside the coronal plain (by the use of the dynamic CT scans), which could therefore not be identified on a plain radiograph (see Fig. 4a and b). A dynamic analysis of a CT scan might improve the visualization of this process because it includes the femoral offset, rotation, version, acetabular coverage and tilt. The calculated sensitivity and specificity however do not highlight these theoretical improvements.

We used the alpha angle and LCE-angle measured on the radiographs. Other radiographic measurements might alternatively be used, e.g. the cross over sign, the posterior wall sign, the version of the hip and more [6]. Using different measurements have advantages and disadvantages. The cross-over sign is mostly used [6] but also no strong evidence exists to support a single best set of radiographic markers for the diagnose of pincer type morphologies [26]. To possibly improve diagnostics, maybe all should be used. Daily practice however still starts with the use of an AP radiograph, for the first impression of a possible pincer morphology/enlarged coverage. An AP radiograph in supine position does also not incorporate the tilt and inclination of the pelvis. In future measurement this could be adjusted by false profile pelvis radiographs and close attention for the pelvic tilt, as very recently stated by Putnam [27].

To use the LCE angle for pincer measurement is debatable. Several diagnostic tests are available for pincer-type FAI. According to Rhee et al. [6], no strong evidence exists to support a single best set of current radiographic markers for the diagnosis of pincer-type FAI. Furthermore, the definition of an enlarged LCE angle is debated and differs from 25 to 40° [28–30]. Rhee et al. [7] describe that most authors use LCE angle greater that 35–40° for acetabular over-coverage, thus pincer morphology. Kutty et al. [10] describe a sensitivity and specificity rate of 84.2 and 100% for an LCE angle of < 40°. These sensitivity and specificity measurements differ with our measurements, which are respectively 82 and 39% for plain radiographs and 84 and 53% for the dynamic CT analyses. A specificity rate of 100% is ideal and might be utopic. These authors have conducted a retrospective study, on a relatively small cohort (55 patients) who were already operated on. This might bias their results and partially explains the differences in our results. Furthermore, these differences could be explained by the use of a different LCE angle.

To compare radiographs with three-dimensional analysis, we used the intra-operative assessment as the golden standard, as stated by Rhee et al. [7]. Specific damage caused to labrum and cartilage could be identified intra-operatively. Beck et al. [23] described how the labrum and cartilage is damaged by cam impingement and by pincer impingement in 244 hips. They described a pattern of damage to the acetabular cartilage and labrum depending on the shape of the hip, induced by repeated microtrauma. Anderson et al. also describes the delamination of acetabular articular cartilage due to femoroacetabular impingement [31]. However, as several patients were diagnosed with only small morphologies, this intra-operative view might be biased by the judgment of the operating surgeon. The surgeon has all pre-operative imaging information. Therefore, if the patient had clinical symptoms of FAI and the imaging revealed a small cam/pincer type morphology, the surgeon might be biased to diagnose an actual morphology if any damage to labrum or cartilage can be identified. It is therefore debatable what gold standard should be used. Other authors have however also used the intra-operative findings as reference standard [25, 32–34].

Positive predictive value for cam type morphology is high for both modalities, but low for pincer type morphology. The negative predictive value is low for cam type morphology but high for pincer type morphology in both modalities. These predictive values seem not very high. As described by Vecchio et al. [35], the predictive values are strongly related to the actual prevalence of the disease (cam/pincer morphology) in the total population. The prevalence of cam morphology in our population was 71% and the prevalence of pincer morphology was 35%. When the prevalence rises, the predictive values grow and are more reliable.

This study has several limitations. Our analysis is retrospective, in a large prospectively registered cohort. Moreover, the analyzed patient population is relatively small. A larger cohort could add more reliable information. Furthermore, we included patients up to 65 years of age, which is relatively high. This however should not influence our diagnostic study design.

The theoretical advantage of the 3D dynamic analysis, the three-dimensional orientation and information about the location and size of the impinging area between acetabulum and femur, could not be highlighted by defining the sensitivity and specificity. These theoretical advantages might improve functional outcome or revision rate, but this is beyond the scope of this article. The extra costs and radiation exposure due to pre-operative CT scans is therefore debatable if it is not improving pre-operative sensitivity and specificity of the diagnostics. The radiation exposure can be bypassed by the use of MRI [36].

## Conclusion

Diagnostic sensitivity, specificity and predictive values are comparable in three-dimensional dynamic analysis of CT scans and radiographs representing FAI caused by cam or pincer type morphology. No clear improvement in diagnostics could be identified with the use of the dynamic analyses, despite that it could assist surgeons in pre-operative planning.

## Data Availability

The datasets used and analyzed during the current study are pubic available from the corresponding author on reasonable request.

## Abbreviations

AP: Antero-posterior
CT: Computerized tomography
FAI: Femoroacetabular impingement
ICC: Intraclass correlation coefficient
LCE: Lateral center edge
METC: Medical ethics committee
MRI: Magnetic resonance imaging
NPV: Negative predictive value
PPV: Positive predictive value
